# A novel mutation conferring the nonbrittle phenotype of cultivated barley

**DOI:** 10.1111/nph.14377

**Published:** 2017-01-16

**Authors:** Peter Civáň, Terence A. Brown

**Affiliations:** ^1^ School of Earth and Environmental SciencesManchester Institute of BiotechnologyUniversity of ManchesterManchesterM1 7DNUK

**Keywords:** agricultural origins, barley, brittle rachis, *Hordeum vulgare*, nonbrittle phenotype

## Abstract

- The nonbrittle rachis, resulting in a seed head which does not shatter at maturity, is one of the key phenotypes that distinguishes domesticated barley from its wild relatives. The phenotype is associated with two loci, *Btr1* and *Btr2*, with all domesticated barleys thought to have either a 1 bp deletion in *Btr1* or an 11 bp deletion in *Btr2*.
- We used a PCR genotyping method with 380 domesticated barley landraces to identify those with the *Btr1* deletion and those with the *Btr2* deletion.
- We discovered two landraces, from Serbia and Greece, that had neither deletion. Instead these landraces possess a novel point mutation in *Btr1*, changing a leucine to a proline in the protein product. We confirmed that plants carrying this mutation have the nonbrittle phenotype and identified wild haplotypes from the Gaziantep region of southeast Turkey as the closest wild relatives of these two landraces.
- The presence of a third mutation conferring the nonbrittle phenotype of domesticated barley shows that the origin of this trait is more complex than previously thought, and is consistent with recent models that view the transition to agriculture in southwest Asia as a protracted and multiregional process.

## Introduction

Cultivated barley (*Hordeum vulgare* L. subsp. *vulgare*), the domesticated form of *Hordeum vulgare* ssp. *spontaneum* (C. Koch), was one of the founder crops of agriculture in the Fertile Crescent of southwest Asia (Zohary *et al*., 2013) and today is the fourth most important in terms of productivity (Ulrich, 2011). Domestication of barley was accompanied by a suite of phenotypic changes brought about by selective pressures resulting from human intervention in propagation of the wild plants (Fuller, 2007). Principal among these changes was an alteration in the architecture of the ears, which results in the mature spikelets (the dispersal propagules containing the grains) remaining attached to the flower head after ripening. This ‘nonbrittle’ phenotype contrasts with the wild ear, which is dehiscent, shattering at maturity and releasing the detached spikelets (Brown *et al*., 2009).

Genetic studies have associated brittleness with two closely linked loci, *brittle‐rachis 1* (*Btr1*) and *brittle‐rachis 2* (*Btr2*) (Takahashi & Hayashi, 1964), which have recently been shown to be independent genes located *c*. 100 kb apart on chromosome 3H (Pourkheirandish *et al*., 2015). Although the genes and their predicted protein products lack sequence or structural similarity, *Btr1* and *Btr2* are functionally related, both specifying a thinning of the cell walls in the rachis node, the structure that attaches the spikelet to the ear (Pourkheirandish *et al*., 2015). As a consequence of this thinning, the mature spikelets of wild plants are able to detach from the ear. The recessive versions of these genes, *btr1* and *btr2*, give rise to rachis nodes with thicker cell walls, which result in a spikelet that can only be detached by threshing. In a recent survey of 240 cultivated barleys, all plants of the *btr1* lineage had the same 1 bp deletion in the *Btr1* gene, and all *btr2* types had the same 11 bp deletion in *Btr2* (Pourkheirandish *et al*., 2015). These results were interpreted as indicating that the *btr1* and *btr2* types of cultivated barley emerged independently in two different geographical regions, *btr1* in the southern Levant and *btr2* somewhat later in the northern Levant.

In this paper we report that some nonbrittle barley landraces have neither the 1 bp deletion in *Btr1* nor the 11 bp deletion in *Btr2* described by Pourkheirandish *et al*. (2015). Instead, these accessions have a point mutation in a different part of the *Btr1* coding sequence. The discovery of this third cultivated lineage has implications for our understanding of the events that resulted in domestication of barley.

## Materials and Methods

### Barley accessions

We studied 380 barley landraces (Supporting Information Table S1), the set covering all ecological and geographic regions of European barley cultivation, and supplemented with a selection of landraces representing African and Asian regions. The set is largely nonoverlapping with the landraces studied by Pourkheirandish *et al*. (2015).

### Genotyping and sequencing the *Btr* loci

We designed rapid genotyping systems capable of distinguishing the dominant and recessive versions of the *Btr1* and *Btr2* genes, assuming the underlying mutations giving rise to the recessive alleles were as previously reported (Pourkheirandish *et al*., 2015). To detect the 1 bp deletion characteristic of *btr1*, we used a PCR in which the 3′‐end of the reverse primer spanned the 1 bp deletion site and produced a 205 bp product from the wild‐type *Btr1* allele and no product from the recessive *btr1* allele. The *Btr2* genotyping system amplified a 154 bp product from the wild‐type allele, and a 143 bp product from *btr2*. Both PCRs were carried out in 12.5 μl reactions comprising 2 ng μl^−1^ DNA, ×1 Q5 reaction buffer, ×1 Q5 high GC enhancer, 0.5 μM each primer, 0.2 mM each dNTP, 0.02 U μl^−1^ Q5 hot start high‐fidelity DNA polymerase (New England BioLabs, Ipswich, MA, USA) with an initial denaturation of 30 s at 98°C, followed by 35 cycles of 10 s at 98°C, 20 s at 66°C, 10 s at 72°C, and a final extension of 2 min at 72°C. Primer sequences are given in Table S2(a).

The status of those landraces that were genotyped as *Btr1 Btr2* according to this screen was checked by sequencing of the *Btr1* and *Btr2* loci. The *Btr1* gene was amplified as a 2425 bp fragment and the *Btr2* gene as a 4942 bp fragment, using the primers described in Table S2(a) and the PCR conditions described earlier, except that for *Btr1* the annealing step was performed at 62°C and the synthesis step was carried out for 1 min, and for *Btr2* annealing was at 60°C and synthesis for 2 min. Amplicons were purified (MinElute PCR Purification Kit; Qiagen) and sequenced (ABI 3730 DNA analyzer; ABI, Foster City, CA, USA) using various primers (Table S2b). Novel sequences have been deposited in GenBank, accession numbers KX722223–KX722226.

### Sequence data analysis

Median joining networks (Bandelt *et al*., 1999) were prepared by aligning the sequences that we obtained with GenBank accessions KR813340–KR813547 (*Btr1/btr1*) and KR813548–KR813810 (*Btr2/btr2*) (Pourkheirandish *et al*., 2015). Alignment gaps were treated as missing data and all polymorphic sites were used in network construction. In these networks, edge lengths are proportional to the number of substitutions, but the node sizes do not reflect haplotype frequencies. Protein Variation Effect Analyzer (Provean) v.1.1 (Choi & Chan, 2015) and the transmembrane orientation of the BTR1 protein predicted by sosui (Hirokawa *et al*., 1998) were used to assess the impact of amino acid changes.

## Results

**Figure 1:**
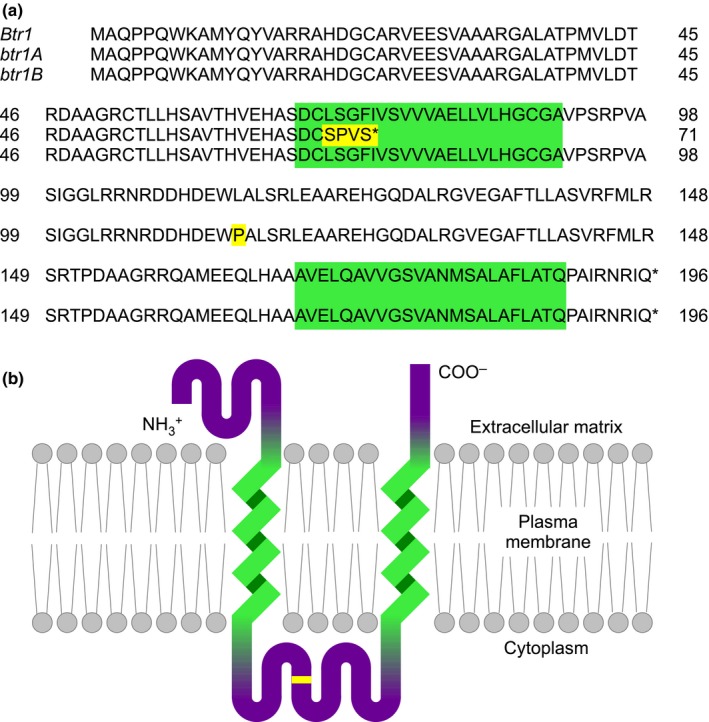
Structure of the novel *btr1* allele in domesticated barley. (a) Alignment between the amino acid sequences of the wild‐type brittle allele (*Btr1*), the previously reported nonbrittle allele (here called *btr1A*), and the novel nonbrittle allele that we report (*btr1B*). The *Btr1* and *btr1A* sequences are from *Hordeum spontaneum* OUH602 and *Hordeum vulgare* cv KNG, respectively (Pourkheirandish *et al*., 2015). The BTR1 protein has two hydrophobic regions indicated by green shading. Changes giving rise to the two *btr1* alleles are highlighted in yellow. (b) Transmembrane orientation of the BTR1 protein predicted by sosui (Hirokawa *et al*., 1998). The hydrophobic regions and the Leu–Pro substitution in the *btr1B* product are indicated using the same colours as in (a).

The genotyping screen identified 300 of the 380 barley accessions as *btr1 Btr2* and 78 as *Btr1 btr2*, each of these accessions having the canonical 1 or 11 bp deletion described by Pourkheirandish *et al*. (2015). The remaining two landraces were typed as *Btr1 Btr2*. These two landraces were PI 374426, collected in 1971 at Raška, Serbia (described as awned, six‐rowed, spring type, hulled), and HOR 683, collected in 1942 from the Peloponnese, Greece (spring type). Sequencing of the *Btr1* and *Btr2* loci in these accessions confirmed that neither possessed the 1 or 11 bp deletions associated with *btr1* and *btr2*, respectively. Instead, both accessions displayed a single T→C transition converting a leucine to proline at position 111 in the *Btr1* protein product (Fig. 1a). Henceforth, we refer to this novel mutation as the *btr1b* allele, in contrast to *btr1a*, which displays the canonical 1 bp deletion.

*Btr1* codes for a transmembrane protein similar to signal transduction receptors. The amino acid substitution specified by the *btr1b* allele is predicted to lie within the cytoplasmic component of the protein (Fig. 1b). provean assigned a score of –5.0 (deleterious) with 95% probability, reflecting the different structural and chemical properties of proline compared with leucine (Wu, 2013). It is therefore reasonable to hypothesize that the substitution will have an impact on the function of the protein.

**Figure 2:**
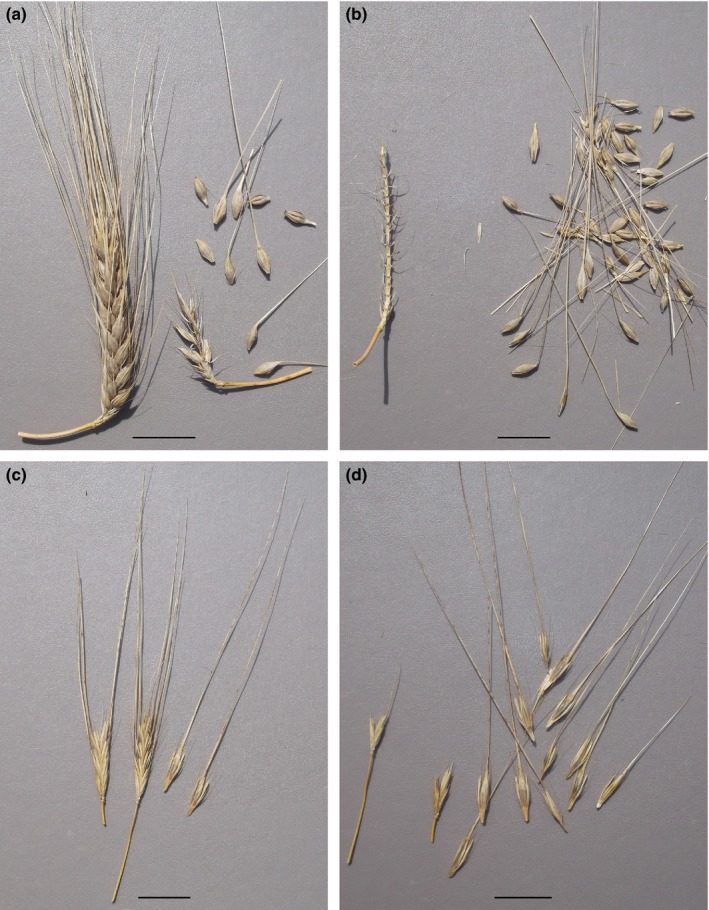
Comparison of the seed head of barley landrace PI 374426, possessing the *btr1b* allele, with a wild barley seed head. (a) Seed head of PI 374426, displaying a nonbrittle, six‐row phenotype. (b) After forcibly removing spikelets (right) from the PI 374426 ear, the rachis remains intact (left). (c) The mature seed head of wild barley accession PI 662202. (d) With PI 662202, the mature rachis disarticulates into segments that remain attached to the spikelets. It is impossible to remove spikelets without breaking the rachis. Bars, 2 cm.

To confirm that the *btr1b* allele confers the nonbrittle phenotype, and that accessions carrying this allele are not mislabeled wild barley accessions, we germinated seeds of PI 374426 and grew plants to maturity. The resulting seed heads had a typical domesticated six‐rowed phenotype (Fig. 2). The mature rachis was tough and remained intact after the spikelets had been forcibly removed from the ear. DNA was then extracted from seeds from two spikelets to confirm the presence of the *btr1b* allele. By contrast, with the wild accession PI 662202, which was genotyped as *Btr1 Btr2*, the mature rachis disarticulated into segments that remained attached to the spikelets, and it was impossible to remove spikelets without breaking the rachis.

Median joining networks were constructed to identify the relationships between the *btr1b* and *Btr2* haplotypes of PI 374426 and HOR 683 and the *Btr1* and *Btr2* haplotypes of wild barley published by Pourkheirandish *et al*. (2015). In the *Btr1*/*btr1* network, the two *btr1b* haplotypes were located together at a position distant from the *btr1a* sequences, and were most closely related (one to four substitutions) to the *Btr1* haplotypes of four brittle wild barleys (IPK IDs FT266, FT624, FT730, FT747), all of which were collected from the Gaziantep region of southeast Turkey (Fig. S1a). The *Btr2*/*btr2* network gave a similar result, the *Btr2* haplotype of PI 374426 and HOR 683 showing the closest relationship with the *Btr2* haplotypes of the same four wild Gaziantep accessions (Fig. S1b).

## Discussion

The assumption that there are two lineages of cultivated barley, originally called the ‘Oriental’ and ‘Occidental’ families, dates back 60 yr to the pioneering genetic studies of Takahashi (1955). Recently, genomic work has suggested that all cultivated accessions fall into one or other lineage, characterized by a 1 bp deletion giving rise to the *btr1* allele and an 11 bp deletion giving *btr2* (Pourkheirandish *et al*., 2015). We show that there is a third type of cultivated barley, whose nonbrittle genotype is conferred by a different mutation in the *Btr1* gene, giving rise to what we have called the *btr1b* allele. Discovery of a third nonbrittle lineage shows that the origins of this phenotype of cultivated barley are more complex than previously thought. Although the novel mutation appears to be scarce in modern landrace collections, this cannot be taken as evidence that it was equally scarce in the past, as the modern frequency of the allele will be, at least in part, a reflection of the vagaries of the postdomestication population dynamics of the crop. Modern landrace collections, which we know to be incomplete records of past diversity, might also under‐represent the historic frequency and geographical distribution of the *btr1b* allele. It cannot therefore be assumed that the emergence of this allele was a ‘minor’ event during the process that led to domestication of barley, nor that the human activities that led to cultivation of this third lineage of domesticated barley were themselves inconsequential. The existence of the novel allele also raises the possibility that other ‘minor’ lineages are as yet undiscovered in germplasm collections, or may have died out during the 10 000 yr since the emergence of the domesticated phenotype.

According to their *Btr1*/*Btr2* haplotypes, the wild accessions most closely related to the *btr1b* domesticates are located in the Gaziantep region of southeast Turkey. Inferring the geographical origin of a domesticated plant lineage by comparison with wild accessions is fraught with danger, even though in this case the wild accessions have been recently collected, and hence have not been affected by gene flow between plants during *in situ* conservation (Jacob *et al*., 2014). Inference of a geographical origin still assumes that the wild phylogeography has been unchanged over the last 10 000 yr, and that the relationship between a domesticate and its source wild population has not been complicated by more recent hybridization between the domesticate and other wild populations. The former assumption is difficult to test, and the latter is almost certainly incorrect (Poets *et al*., 2015). However, we note that those wild accessions with *Btr2* haplotypes closest to *btr2* come from northern Syria and the Gaziantep area of Turkey (Pourkheirandish *et al*., 2015). It is therefore possible that the domesticated lineages with the *btr2* and *btr1b* alleles derive from the same wild population source.

Hypotheses regarding the origins of agriculture in the Fertile Crescent of southwest Asia have undergone a dramatic shift in recent years. The earlier interpretation of the transition from hunter‐gathering to agriculture as a rapid ‘revolutionary’ event, with each of the founder crops of agriculture emerging from one or, at most, two discrete geographical locations (Diamond, 2002), is now being replaced by a more sophisticated model in which the transition to agriculture was a protracted and multiregional process, with the domesticated germplasm of each crop derived from a variety of wild populations (Civáň *et al*., 2013; Willcox, 2013; Poets *et al*., 2015). The presence of additional brittle rachis mutations in cultivated barley is consistent with this model and emphasizes the geographically dispersed nature of the anthropological and microevolutionary events that resulted in the emergence of agriculture in southwest Asia.

## Author contributions

P.C. carried out the laboratory work and data analysis. P.C. and T.A.B. jointly conceived the project and wrote the manuscript.

## Supporting information

Please note: Wiley Blackwell are not responsible for the content or functionality of any Supporting Information supplied by the authors. Any queries (other than missing material) should be directed to the *New Phytologist* Central Office.

**Fig. S1** Median‐joining networks showing the relationships between the cultivated accessions containing *btr1b* alleles and wild barley accessions.

**Table S1** Barley landraces.

**Table S2** Primers: PCR primers and sequencing primers.
