# Supplementary material for: A novel mutation conferring the nonbrittle phenotype of cultivated barley
Source: New Phytol. 2017 Jan 16;214(1):468–72. doi: 10.1111/nph.14377 (PMC5347957; doi:10.1111/nph.14377)

New Phytologist

***New Phytologist* Supporting Information**

Article title: **A novel mutation conferring the non-brittle phenotype of cultivated barley**

Authors: Peter Civáň and Terence A. Brown

Article acceptance date: 3 November 2016

The following Supporting Information is available for this article:

Fig. S1 Median-joining networks showing the relationships between the cultivated accessions containing *btr1b* alleles and wild barley accessions.

Table S1 Barley landraces. (See separate file.)

Table S2 Primers: (a) PCR primers; (b) sequencing primers. (See separate file.)

New Phytologist

Fig. S1 Median-joining networks showing the relationships between the cultivated accessions containing *btr1b* alleles and wild barley accessions. Alignment gaps were treated as missing data and all polymorphic sites were used. Node lengths are proportional to the number of substitutions, but the node sizes do not reflect haplotype frequencies. (a) Network prepared from an alignment of *Btr1* haplotypes (GenBank: KR813340–KR813547) supplemented with the *btr1b* haplotypes present in PI 374426 and HOR 683. Nodes containing domesticated barley are coloured in blue. The *btr1b* haplotype is most closely related (1–4 substitutions) to four brittle wild barleys (IPK IDs FT266, FT624, FT730, FT747), all of which were sampled around Gaziantep, southeast Turkey. (b) Network prepared from an alignment of *Btr2* haplotypes (GenBank: KR813548–KR813810) supplemented with the *Btr2* sequences obtained from PI 374426 and HOR 683. The latter two *Btr2* haplotypes form a single node most closely related to the same four brittle wild barleys (circled).

(a)

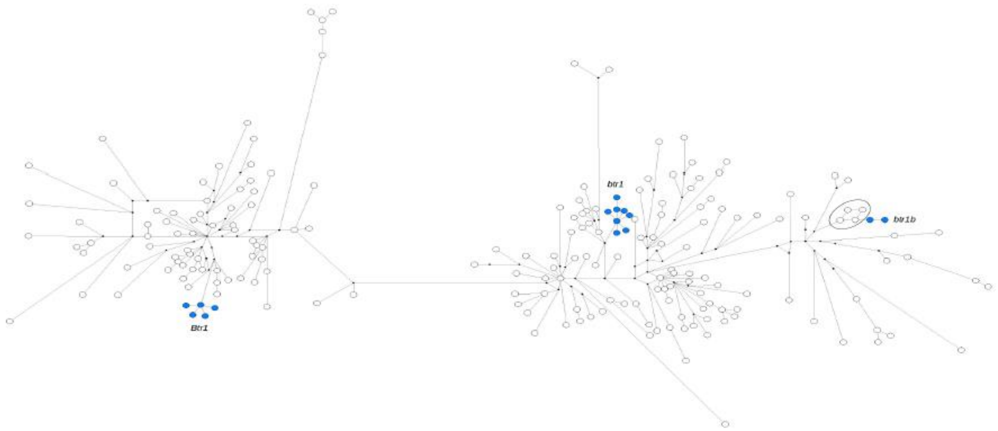

(b)

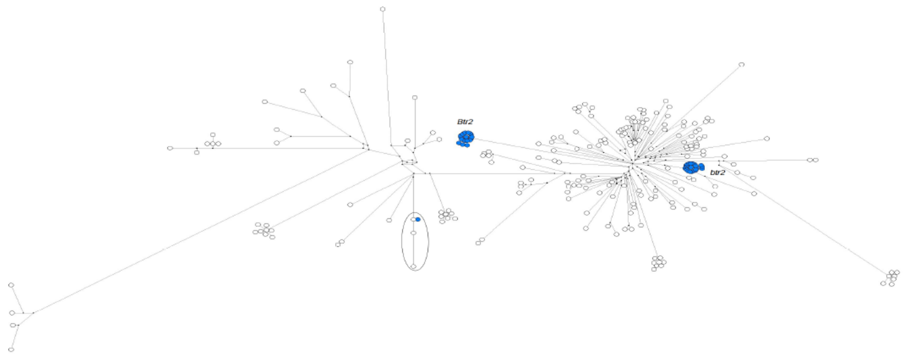

Supplement: Supplementary file 1 — Fig. S1 Median‐joining networks showing the relationships between the cultivated accessions containing btr1b alleles and wild barley accessions. [file NPH-214-468-s001.pdf]
